# Macroscale intraspecific variation and environmental heterogeneity: analysis of cold and warm zone abundance, mortality, and regeneration distributions of four eastern US tree species

**DOI:** 10.1002/ece3.1752

**Published:** 2015-10-16

**Authors:** Anantha M. Prasad

**Affiliations:** ^1^ Northern Research StationUSDA Forest Service359, Main RoadDelawareOhio43015

**Keywords:** Climate change, climatic zones, decision‐tree‐based ensemble methods, eco‐evolutionary processes, Forest Inventory Analysis, intraspecific variation, local adaptation, plant hardiness zones

## Abstract

I test for macroscale intraspecific variation of abundance, mortality, and regeneration of four eastern US tree species (*Tsuga canadensis*,*Betula lenta*,*Liriodendron tulipifera,* and *Quercus prinus*) by splitting them into three climatic zones based on plant hardiness zones (PHZs). The primary goals of the analysis are to assess the differences in environmental heterogeneity and demographic responses among climatic zones, map regional species groups based on decision tree rules, and evaluate univariate and multivariate patterns of species demography with respect to environmental variables. I use the Forest Inventory Analysis (FIA) data to derive abundance, mortality, and regeneration indices and split the range into three climatic zones based on USDA PHZs: (1) cold adapted, leading region; (2) middle, well‐adapted region; and (3) warm adapted, trailing region. I employ decision tree ensemble methods to assess the importance of environmental predictors on the abundance of the species between the cold and warm zones and map zonal variations in species groups. Multivariate regression trees are used to simultaneously explore abundance, mortality, and regeneration in tandem to assess species vulnerability. Analyses point to the relative importance of climate in the warm adapted, trailing zone (especially moisture) compared to the cold adapted, leading zone. Higher mortality and lower regeneration patterns in the warm trailing zone point to its vulnerability to growing season temperature and precipitation changes that could figure more prominently in the future. This study highlights the need to account for intraspecific variation of demography in order to understand environmental heterogeneity and differential adaptation. It provides a methodology for assessing the vulnerability of tree species by delineating climatic zones based on easily available PHZ data, and FIA derived abundance, mortality, and regeneration indices as a proxy for overall growth and fitness. Based on decision tree rules, ecologically meaningful variations in species abundance among the climatic zones can be related to environmental variability and mapped.

## Introduction

Species are composed of populations that are spatially differentiated due to temporally varying interactions and feedbacks between their genotypes and the local environment in which they live (Kawecki and Ebert 2004; Post and Palkovacs 2009; Shaw and Etterson 2012). These genotype × environment feedbacks and the resulting reaction norms (Lewontin 2006) can result in local adaptation that manifests itself as intraspecific variation. This variation is particularly important in trees because they have long generation times, large population sizes, and harbor large genetic diversity and gene flow due to high fecundity (Petit and Hampe 2006). Some tree seeds also have large dispersal distances (Aitken et al. 2008) and exhibit considerable plasticity in their phenotypic response (Nicotra et al. 2010). These traits, along with the fact that many hybridize (Hoffmann and Sgrò 2011) and can adapt, modify, and construct their niches (Levins 1979; Day and Laland 2003), make trees especially challenging to model under changing environmental conditions.

Since trees first appeared in the middle to late Devonian Period, 380 million years ago (about 325 mya for conifers and 140 mya for angiosperms), they have evolved in response to extreme climatic fluctuations, by diversifying their taxa – via tracking, conserving, and expanding their niches (Hamrick 2004; Pearman et al. 2008), adapting to and changing their local environment, or even becoming extinct in some cases (Jackson and Weng 1999). These eco‐evolutionary feedbacks in deep time, coupled with environmental changes, have resulted in many genetic and phenotypic patterns of spatially varying adaptation within species (Davis et al. 2005). In addition to climate, tree populations can be locally adapted to edaphic and biotic factors (Savolainen and Bokma 2007; Savolainen et al. 2007). Some of the variation could be due to stochasticity (i.e., genetic drift), and past rather than present selective forces (Aizen and Woodcock 1992). Because of their sessile nature, trees exhibit pronounced phenotypic plasticity, whereby a single genotype can respond to changes in the environment by rendering different phenotypes, some of which can be adaptive (Via and Lande 1985; Pigliucci et al. 2006; Nicotra et al. 2010). There are, however, limits to phenotypic plasticity due to ecological interactions and physiological bottlenecks which can constrain trees’ adaptive potential (Valladares et al. 2007). In spite of the extensive knowledge of natural and evolutionary history of trees, there is much uncertainty about how trees will respond to rapid climate change described by the Intergovernmental Panel on Climate Change (IPCC, 2013). Also, even though there may be enough standing genetic variation in trees to adapt to rapid climate change without the need for new mutations, it is doubtful whether ecological and developmental constraints to selection can be overcome (Lewontin 2003).

From an evolutionary standpoint, natural selection has been known to result in locally adapted phenotypes (Aitken 2004). However, the resulting genetic variation within and among populations can be modified by mutation, gene flow, genetic drift (especially in smaller populations), hybridization, and recombination (Holt and Gomulkiewicz 1997; Soltis and Soltis 2009). Gradients in the form of ecoclines can exist due to geographic barriers and features, life history, frequency, and density‐dependent selection, and hence can render populations to suboptimal ecological conditions (Rehfeldt and Ying 1999; Rehfeldt et al. 2001). Furthermore, some populations may be in climatic disequilibrium due to evolutionary lag in spatial niche tracking or dispersal constraints (Araújo and Pearson 2005; Sexton et al. 2009). Anthropogenic climate change will likely render tree populations maladapted compared to other abiotic factors (IPCC 2013). Adaptation to climate change will depend on phenotypic traits relevant in the new environments, such as timing of growth and tolerance to seasonal drought or cold (Alberto et al. 2013) that vary spatially within and among populations. For example, many oaks in eastern North America are currently adapted to drought‐prone sites (Abrams 1990) and their suitable habitats can change depending on future changes in temperature and precipitation as well as biotic interactions (Prasad et al. 2013). Because of eco‐evolutionary processes that result in intraspecific variation, populations are likely to respond differentially to rapid changes in climate and other stressors compared to the species as a whole (Rehfeldt and Ying 1999; Pearman et al. 2010); this effect is likely to be more pronounced in the edges of the species range (Geber 2008).

It is therefore clear that there is considerable complexity due to genetic, environmental, and developmental factors which result in intraspecific variation of tree species. It is very difficult to disentangle the contribution of different factors in any specific study. However, this intraspecific variation needs to be better recognized in species habitat models to reduce the tendency to show anomalous responses and exaggerated extinction risk (Morin and Thuiller 2009). Until recently, most habitat distribution models of tree species assumed that species are genetically homogeneous across their entire range, exhibiting similar adaptation and plasticity (Alberto et al. 2013). This assumption is being questioned, with some researchers taking a population‐based approach, thereby recognizing intraspecific variation (O'Neill et al. 2008; Garzón et al. 2011; Banta et al. 2012; Oney et al. 2013; Pironon et al. 2015; Slaton 2015).

Frequently, the data for intraspecific variation have been derived from provenance studies and common garden experiments (Carter 1996; Mátyás 1996). These studies offer a wealth of information on quantitative genetics and population differentiation due to selection, plasticity, gene flow, and genetic drift (Kremer et al. 2012), but are limited to a few commercially important tree species and were not established for the evaluation of potential climate change (Wang et al. 2010; Leites et al. 2012). Therefore, there is a need to use more commonly available data to assess range‐wide intraspecific variation of tree species due to environmental heterogeneity. While it is desirable to include genetic variation, this requires neutral as well as adaptive genetic markers which are not easily available for multiple species. Nevertheless, exploring environmental heterogeneity in intraspecific variation is worthwhile especially if the results can show ecologically meaningful patterns.

### Objectives

The principal aim of this paper was to explore macroscale intraspecific variation of the demographic variables (abundance, mortality, and regeneration) of four eastern US tree species and analyze how they are related to environmental heterogeneity via climatic, edaphic, and topographic variables. I assess intraspecific variation, treating each species as differentially adapted along climatic and geographic space without the complexity of genetic subspecies differentiation or provenance studies (Newton et al. 1999). In the process, I analyze how environmental variability within and among broadly defined climatic zones can affect abundance, mortality, and regeneration measures, and specifically investigate how the cold adapted, leading zone of the range differs from the warm adapted, trailing zone. I use the term leading and trailing to indicate positions in climatic‐geographic space and not movement or migratory potential of the species. In addition, I explore the multivariate response of abundance, mortality, and seedling count (SC) together as a group, in association with environmental variables.

The purpose of this paper was not to predict newer habitats based on regional variations in abundance, but rather to unravel ecologically meaningful patterns of intraspecific variation of demographic variables, and also to describe a methodology for identifying and extracting useful, regional variations in abundance via decision tree rules.

## Methods

For deriving demographic data, 137,704 Forest Inventory Analysis (FIA) plots (Smith 2002; Woudenberg and Conkling 2010) in the eastern United States were used to derive importance value (IV), percent mortality (PM), and SC and aggregated them to 10‐km cells. This was done for the four tree species (*Tsuga canadensis*,* Betula lenta, Liriodendron tulipifera,* and *Quercus prinus*). IV, a measure of relativized abundance, is calculated from the basal area and number of stems of the overstory and understory of the species, and incorporates the biotic influence of other species within the plot (Iverson and Prasad 1998; Iverson et al. 2008). IV is calculated as:

$$\text{IV}\left(x\right)=\;\frac{50*\text{BA}(x)}{\Sigma \text{BA}(\text{all species in plot})}\;+\;\frac{50*\text{NS}(x)}{\Sigma \text{NS}(\text{all species in plot})}$$

where *x* is a particular species in a plot, BA is the basal area, and NS is the number of stems (summed for overstory and understory trees). In monotypic stands, the IV would reach a maximum of 100. IV was used as a surrogate for growth and survival and provides a univariate measure of “fitness” in the realized niche. I also derived PM based on whether the sampled tree is alive or dead in the plot, and SC (number of seedlings < 1 inch in diameter and at least 12 inches tall) from the FIA data for these three zones. These three demographic measures together provide a rough measure of what I refer to as the “overall fitness” of the species (Nagaraju et al. 2013).

I used USDA's plant hardiness zone (PHZ) data (U.S. Department of Agriculture, 2012), which uses average annual extreme minimum temperatures from 1976 to 2006, to split IV for the four tree species into three zones roughly delineating cold adapted, leading region referred from now on as CLR, middle, well‐adapted core region (MCR), and warm adapted, trailing region (WTR).

**Figure 1:**
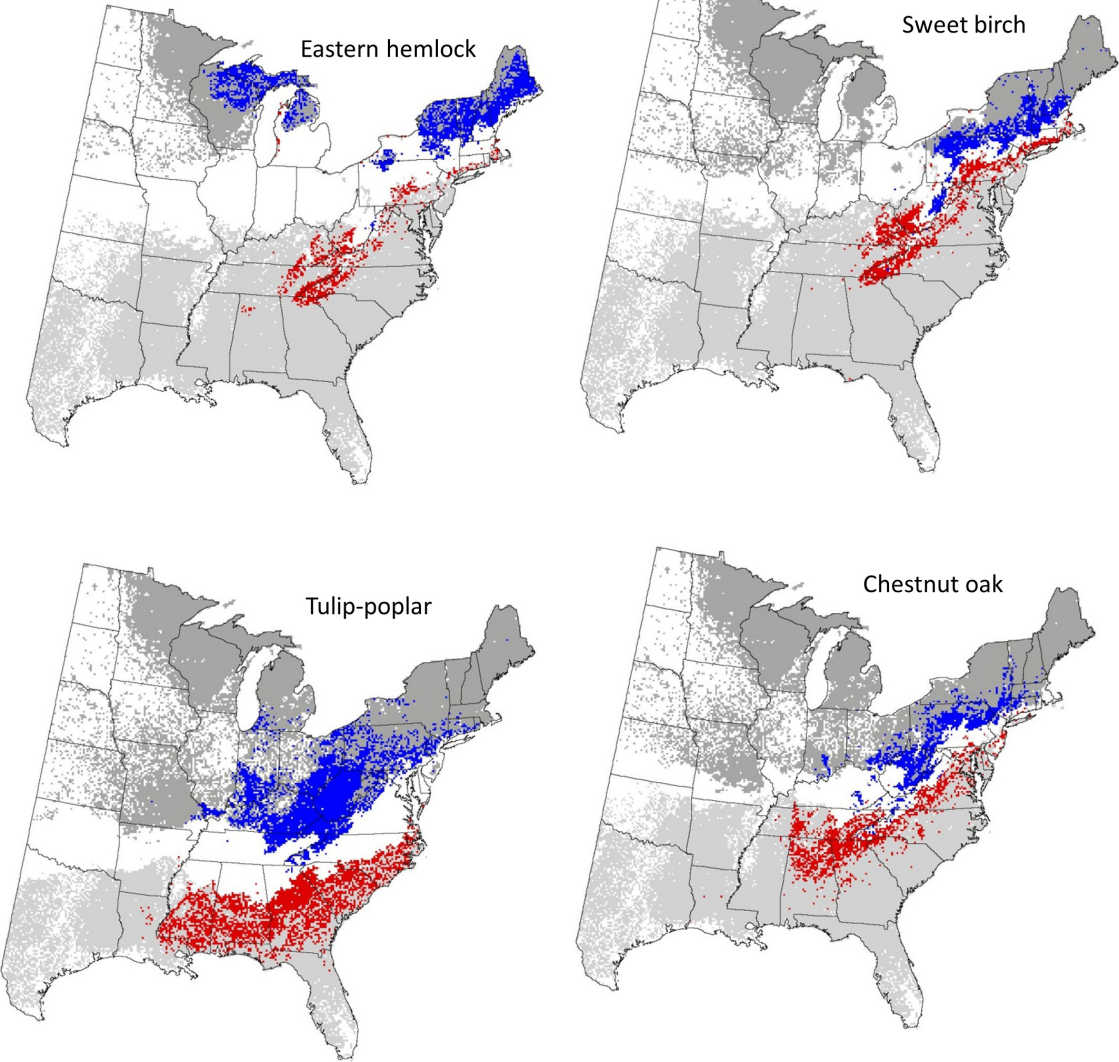
The distribution of the four species in the cold (blue) and warm (red) zones. The dark gray depicts cells with Forest Inventory Analysis (FIA) plots where the species is absent in the cold zone, and the light gray depicts cells with FIA plots where the species is absent in the warm zone.

Because PHZs use average annual extreme minimum temperatures (1976–2006) to split the continental United States into 19 zones, they provide a proxy for cold tolerance of the species and were a convenient way to climatically split the geographic space (average annual extreme minimum temperatures varies from −42.8°C to 10°C) of each species represented by IV (Howe and Aitken 2003). The PHZ boundaries often represent climatic range limits for many tree species (Vogel et al. 2005; Bower et al. 2014). The delineation into the three climatic zones was based on the iterative assessment of histogram and quartile information depending on how the PHZs defined by the USDA varied for the species, as well as visual inspection of the intersection of PHZs and IV of the species in a GIS (Figure 1 and Table 1).

**Table 1:** The average annual extreme minimum temperatures and the corresponding plant hardiness zones (in parenthesis) for each of the three zones for the four species

Species name	Cold leading zone (CLR)	Middle core zone (MCR)	Warm trailing zone (WTR)
Eastern hemlock	−37.2 to −26.1°C (6–9)	−26.1 to −20.6°C (10–11)	−20.6 to −9.5°C (12–15)
Sweet birch	−37.2 to −26.1°C (6–10)	−23.3 to −20.6°C (11)	−20.6 to −3.9°C (12–17)
Tulip poplar	−31.7 to −17.8°C (8–12)	−17.8 to −12.2°C (13–14)	−12.2 to −3.9°C (15–17)
Chestnut oak	−31.7 to −20.6°C (8–11)	−20.6 to −17.8°C (12)	−17.8 to −3.9°C (13–17)

A parsimonious set of nine ecologically meaningful explanatory variables were derived after screening for multicollinearity and relevance (via multiple model runs). These were aggregated at 10 km and also 4 km to derive finer scale maps from rules based on 10‐km model (as explained later in this section).

The predictor set included (abbreviations follow):

1. Three seasonal climate variables based on PRISM data (PRISM Climate Group 2004) spanning from 1981 to 2010 for the current climate (originally at 4‐km resolution):
  - Growing season aridity index (*gsai*) – a ratio of total May to September precipitation to mean May to September potential evapotranspiration.
  - Growing season average temperature (*tmaysep*) – May to September average temperature.
  - Growing season average precipitation (*pmaysep*) – May to September average precipitation.

The correlation among climatic variables was not high: between gsai and tmaysep, it was −0.27; tmaysep and pmaysep, 0.28; gsai and pmaysep, < −0.01.

Five edaphic variables based on Soil Survey Geographic (SSURGO) data (Peters et al. 2013):
  - Soil pH (*ph*)
  - Percentage coarse texture (> 2 mm) (*sieve10*),
  - Percent fine texture (<0.074 mm) (*sieve200*),
  - Percent clay (<0.002 mm) (*clay*),
  - Soil productivity (*sprod*).

Soil productivity index (*sprod*) is an ordinal measure derived from family‐level soil taxonomy information. Soils are ranked from 0 (least productive) to 19 (most productive) based on organic matter content, cation exchange capacity, and percent clay (Schaetzl et al. 2012).

One topographic variable based on US Geological Survey, 30 m SRTM data (Farr and Kobrick 2000):
  - Maximum elevation in meters (*elvmax*).

The predictors are described in Table 2.

**Table 2:** The predictors used in the decision tree based ensemble models. The cell resolution is 10 km for the models and 4 km for mapping decision tree rules

Predictor abbreviation	Description
*gsai*	Growing season aridity index: Ratio of total May to September precipitation to mean May to September potential evapotranspiration
*tmaysep*	Growing season average temperature: May to September average temperature, °Celsius
*pmaysep*	Growing season average precipitation: May to September average precipitation, mm
*ph*	Soil pH
*sieve10*	Percentage coarse texture (>2 mm)
*sieve200*	Percent fine texture (<0.074 mm)
*clay*	Percent clay (<0.002 mm)
*sprod*	Soil productivity: ordinal measure derived from family‐level soil taxonomy information. Soils are ranked from 0 (least productive) to 19 (most productive) based on organic matter content, cation exchange capacity, and percent clay
*elvmax*	Maximum elevation in meters, US Geological Survey, 30 m SRTM data

Because the zones were delineated using average annual extreme minimum temperatures, additional temperature‐related variables like minimum January temperature were not necessary. *Gsai* gives a measure of growing season moisture stress that is predicted to become increasingly important in the future because evapotranspiration is forecast to increase more than precipitation in some regions, increasing moisture stress of various ecosystems (Barber et al. 2000; Breshears et al. 2005; McDowell et al. 2008; Lindner et al. 2010). The *tmaysep* variable represents growing season temperature/heat stress that is currently important and is likely to become more important in the future for some species (Kapeller et al. 2012). *Pmaysep* is important as a direct indicator of growing season water availability.

Also, while elevation and temperature are generally correlated, in the eastern United States, the correlation between *elvmax* and *tmaysep* is not high (−0.55). Therefore, it is assumed that there are other associated ecological and environmental factors that make *elvmax* a useful explanatory variable for many species.

The IV of the four species was split into three PHZs as shown in Figure 1. The PHZs and temperatures for each of the zones are listed in Table 1. All pixels that had FIA plots are included in the three zones, including those of nonpresence in order to delineate the entire climatic space as defined by the PHZs. The geographic space generally matches the climatic space because climate generally follows latitudinal gradients; however, there can be differences due to topography and the presence of water bodies (e.g., the borders of the Great Lakes tend to fall in warmer PHZ).

It should be borne in mind that the geographic area delineating climatic zones is based approximately on cold tolerance and may not be correlated with genetic distance (Wilkinson 2001).

### Species selection

The four species chosen to explore intraspecific variation – eastern hemlock (*T. canadensis*)*,* sweet birch (*B. lenta*), tulip poplar (*L. tulipifera*), and chestnut oak (*Q. prinus*) – are present throughout much of the eastern US forests. Of the four, eastern hemlock is the only one that extends into Canada and was chosen because it is currently under threat of hemlock woolly adelgid (HWA) (Potter et al. 2007, 2011) . The distribution of sweet birch and chestnut oak are worth studying because their climatic gradient spans both north–south and east–west in the Appalachian corridor. Tulip poplar spans a wider region from west to east as well as from north to south and was chosen to be representative of a species with a large N‐S environmental gradient. The four species thus span heterogeneous environmental gradients and are representative of many important tree species in eastern United States.

### Statistical methods and analysis

I evaluate the relationship between abundance (IV) and ecologically relevant environmental variables using decision‐tree‐based techniques. Ensemble techniques based on decision trees have consistently proven to be reliable in data analysis with complex interactions and nonlinear structure (Lawler et al. 2006; Prasad et al. 2006; Park and Chon 2007). These techniques have been previously used to predict and map changes in tree species habitats (Iverson et al. 2008) and assess colonization likelihoods of these suitable habitats (Iverson et al. 2004; Prasad et al. 2013) in the eastern United States, using all or most of the range of the species.

### Univariate analysis

To assess the changes in the importance of environmental variables in the different zones, random forest (RF) models (Prasad et al. 2006; Cutler et al. 2007) using IV as their response were used to assess model fit and the importance of the explanatory variables (randomForest package in R) (R Development Core Team 2014). I grouped the variable importance scores as estimated by RF model (1000 trees were evaluated after automating the selection of the best subset of predictors based on 750 runs) into climate (*gsai*,* tmaysep,* and *pmaysep*), soil (*ph*,* clay*,* sieve10*, and *sprod*) and topographic (*elvmax sieve200*) categories in order to bring out the differences among these categories for CLR and WTR. Also, within the climate category, I differentiated between gs_moisture (*gsai* and *pmaysep*) and gs_temperature (*tmaysep*) in order to highlight the differences between them as these can figure prominently under future climates.

A pruned decision tree model with eight terminal nodes was used for grouping abundance classes using rules based on environmental variables (rpart in R). The reason eight nodes were chosen (as opposed to a smaller number) was for practical reasons – mainly interpretability within a page, while not sacrificing nuances in the terminal nodes. I developed the decision tree rules for IV using a 10‐km resolution model and extracted species abundance groups using these rules from predictors and abundances at 4‐km resolution. Using macroscale rules to extract groups based on finer scale predictors is useful when we suspect that lower branches depict finer scale processes as it often does in regression trees (Iverson and Prasad 1998).

I also compared the IV, PM, and SC indices to gain insight into macro‐ecological differentiation between the CLR and the WTR zones. Statistical significance of the differences in the mean value between the cold and warm zones was determined using Student's *t*‐test (after taking a random sample of 100 from the statistical population and using log transformation for normalization). To implement the correct *t*‐test, the *F*‐test was used to determine whether the population variances were the same. *Gsai* and *pmaysep* were used as surrogates for moisture stress and *tmaysep* as surrogate for temperature stress, because these variables could act within and among populations to separate out climatic clines. Because tree species also differentiate along edaphic and topographic clines, the relative importance of climate, soil, and topography was evaluated.

### Multivariate analysis

The combined demographic response of the species would include abundance, mortality, and regeneration measures, which in tandem approximately correspond to the “overall fitness.” Exploring the multivariate response with respect to environmental variables is a useful heuristic exercise and can point to some unexpected associations that can be probed further. I used multivariate regression trees (mvpart package in R) to explore “overall fitness” and associate them with environmental variables.

Multivariate regression trees is a form of regression where in addition to the prediction of responses, the results can be interpreted as a form of constrained clustering, yielding similar clusters defined by a set of environmental variables. The clusters define the assemblage and environmental values define the associated habitat type as in the conventional community analysis (De'Ath 2002).

The goal of the multivariate analysis was to investigate macroscale ecological differences and similarities between CLR and WTR.

## Results

**Figure 2:**
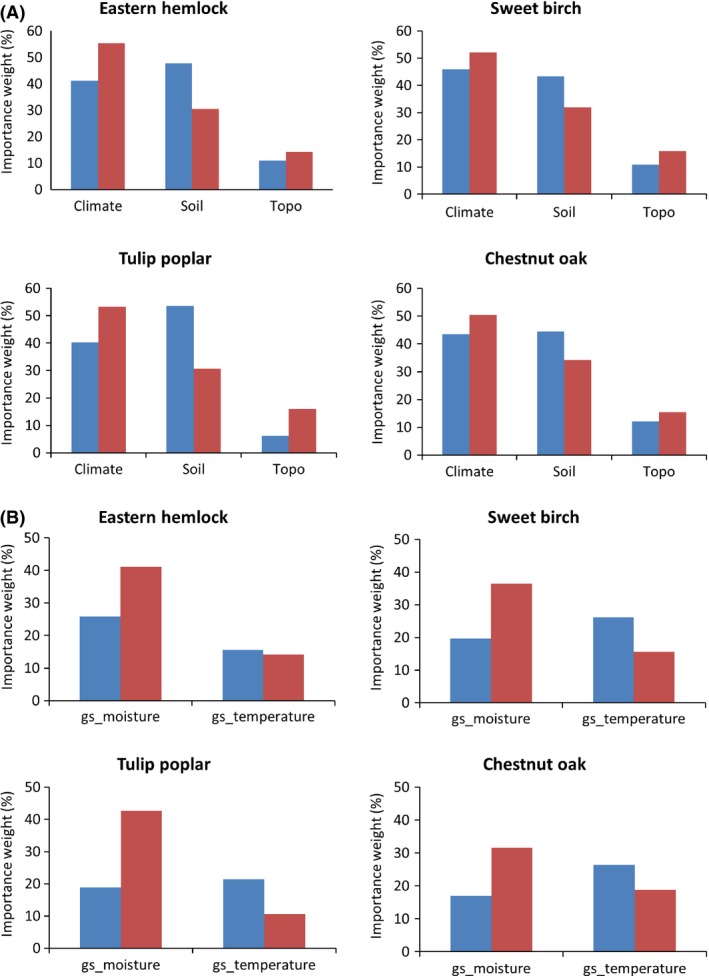
The importance scores (percent importance weight on the y axis) of the Random Forest model. (A) Climate (tmaysep, pmaysep and gsai), soil (sieve10, sieve100, ph, clay and sprod), and topographic (elvmax) predictors between the cold and the warm zone. (B) Splits the climatic component to growing season moisture (gs_moisture: gsai and pmaysep) and temperature (gs_temperature: tmaysep) in order to highlight the importance of these two components. Blue is cold zone and red is warm zone. See Table 2 for description of the predictors.

I will confine the results to just CLR and WTR zones in order to highlight the differences between these two fronts. As is evident from Figure 2(A), in all four species, the importance of climate (as compared to soil and topo) increases in the WTR. And more importantly, within the climate component, the importance of growing season moisture (gs_moisture: gsai and pmaysep) as compared to growing season temperature (gs_temperature: tmaysep) increases in the WTR compared to the CTR (Figure 2B). The importance of climate in the warm zone is most prominent for tulip poplar and eastern hemlock. The gs_moisture component in WTR is most prominent for eastern hemlock, sweet birch, and tulip poplar and somewhat less so for chestnut oak. We see a general trend wherein the climate component, and especially the gs_moisture component, becomes important in the WTR compared to the CLR. This has some important implications under future climate where the differences in growing season temperature and moisture are expected to become important in determining the species response (Adams et al. 2009; Allen et al. 2010; Anderegg et al. 2012). The RF model fit (*R* ^2^) was acceptable and varied between 0.58 to 0.36 for the cold zone and 0.39 to 0.24 for the warm zone.

**Figure 3:**
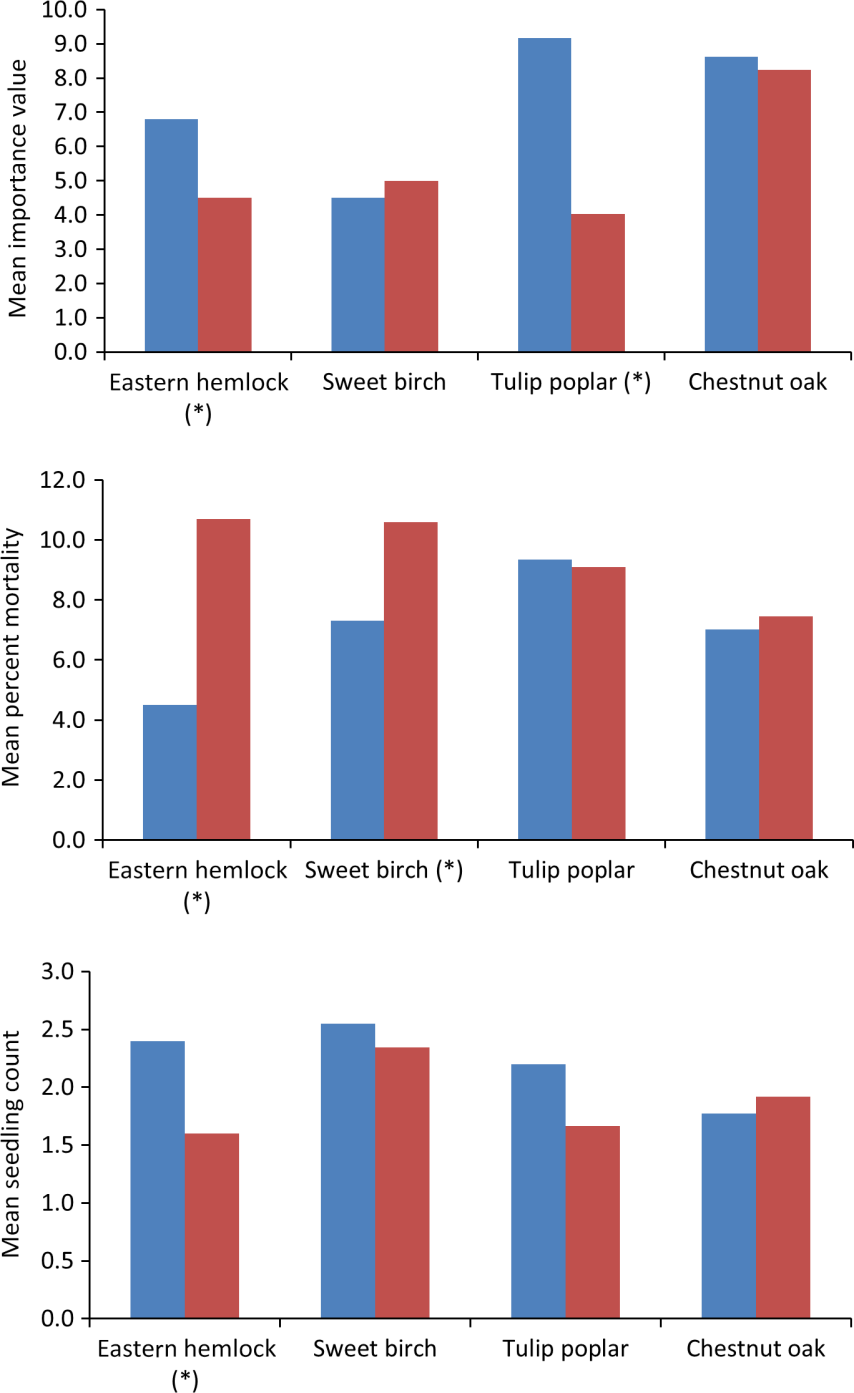
The population means of responses (Importance value; percent mortality; seedling count) between the cold and warm zones (for values greater than zero). The asterisk in parenthesis shows that the difference between the two zones is statistically significant (at 95%) in the *t*‐tests. Blue is cold zone and red is warm zone.

Comparing the three responses (IV, PM, and SC) separately between the cold and warm zones (Figure 3) reveals that only eastern hemlock shows significant difference between the zones. The IV of tulip poplar and PM of sweet birch are the only other results that are statistically significant. For eastern hemlock and sweet birch, the PM increases in the warm zone which along with a decreased SC points to vulnerability. For eastern hemlock, this could be because of the HWA, which appears to have a cold limit that prevents it from moving into the northern part of its range much (Dukes et al. 2009). Also, there is a pattern where the SC is higher in the CLR compared to the WTR (chestnut oak being the exception). Except for sweet birch, the IV is lower in the WTR and is significantly different only for eastern hemlock and tulip poplar. Again, the fact that mortality in general tends to increase in the WTR along with decreased regeneration points to increasing vulnerability of the species in the warm trailing region.

### Rule‐based regional species group extraction

**Figure 4:**
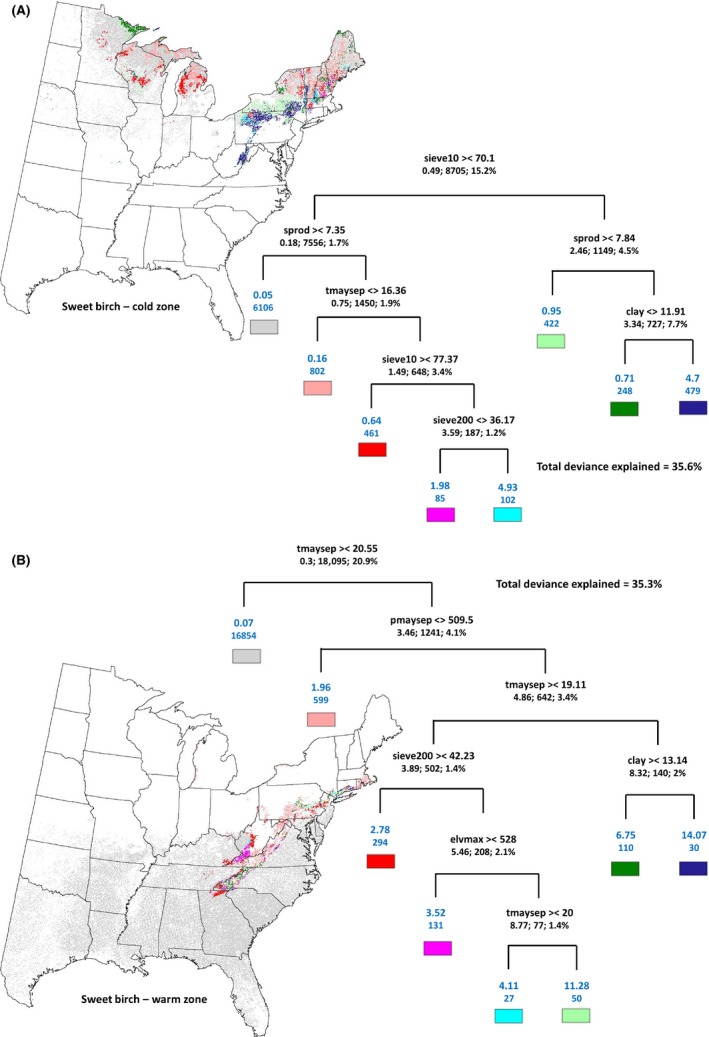
The map corresponding to the rule sets for cold zone (A) and warm zone (B) for sweet birch depicted by the eight‐node decision tree. The colors at the terminal nodes are the legend for the map and can be traced by traversing the tree from the top node. The numbers at the terminal node are the mean IV (top) and number of pixels (bottom) for that node. The numbers below the rules in the nonterminal nodes are as follows: mean IV; number of pixels; % deviance explained. The total deviance explained by the pruned decision tree is also shown. See Table 2 for description of the predictors. The rule‐based maps for the rest of the species are in the Supporting information.

A key feature of the zonal analysis is the ability to extract regional groups of species using decision tree rules developed at 10‐km resolution and mapping the rule set based on predictors at 4‐km resolution. Using sweet birch as an example, I illustrate how an eight‐node pruned decision tree can be used to delineate these species abundance groups according to the rules depicted by the branches of the decision tree for CLR and WTR (Figure 4A and B).

As an example, if our goal is to identify and map regions with high abundance (IV) in the cold zone of sweet birch (Figure 4A), the terminal nodes corresponding to IV of 4.93 and 4.7 are the regions to extract. The corresponding rules for the terminal node with IV of 4.93 are as follows: sieve10 > 70.1% and sprod < 7.35 and tmaysep > 16.36°C and sieve10 < 77.37% and sieve200 > 36.17%. This branch has been mapped as cyan‐colored class in the corresponding map. The rule set for terminal node with IV of 4.7 is as follows: sieve < 70.1% and sprod < 7.84 and clay > 11.91%. This branch has been depicted as dark blue in the map. Both set of rules have strong soil component in the cold zone showing that high abundance in the leading region need not always be climate‐driven.

In the warm zone (Figure 4B), the two terminal nodes corresponding to high IV are 11.28 and 14.07. The warmer zone, even though smaller in range, does show higher values of IV compared to the cold zone for sweet birch. The rules for terminal node with IV of 11.28 are as follows: tmaysep < 20.55°C and pmaysep > 509.5 mm and tmaysep > 19.11°C and sieve200 < 42.23% and elvmax < 528 m and tmaysep < 20°C. This branch has been mapped as light green‐colored class in the corresponding map. The rule set for terminal node with IV of 14.07 is as follows: tmaysep < 20.55°C and pmaysep > 509.5 mm and tmaysep < 19.11°C and clay < 13.14%. This branch has been depicted as dark blue in the map. For sweet birch, climate figures prominently in the warm zone compared to the cold zone. The maps and decision trees for the other species are in the Supporting information.

### Geographic versus environmental space

I also wanted to test how much the inclusion of latitude and longitude of the pixels as explanatory variables (i.e., the macroscale spatial trend) improved the fit. A large increase would mean macroscale spatial autocorrelation is important and the geographic gradient competes with the environmental gradient in explaining the trends in the models. However, the inclusion of latitude and longitude only improved the model results marginally (*R*‐square never increased by more than 0.04 and averaged around 0.02). This suggests that environmental distance is more important compared to geographic distance in explaining the variation of response in this study.

### Multivariate analysis

**Figure 5:**
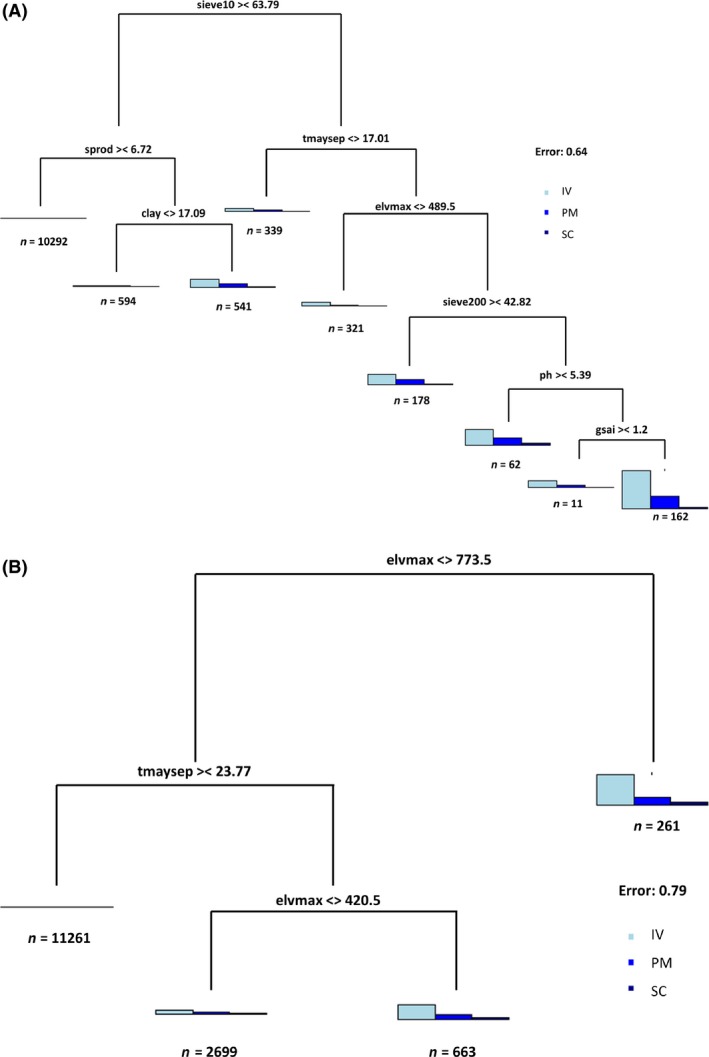
The multivariate decision tree for the cold zone (A) and warm zone (B) of chestnut oak. One can trace the rules that predict different combinations of importance value (IV), percent mortality (PM), and seedling count (SC) together. Under each histogram depicting the multivariate response, *n* is the number of pixels in the node. The length of the lines corresponds to the importance of the branch in explaining the deviation. See Table 2 for description of the predictors. The results of the multivariate analysis for the rest of the species are in the Supporting information.

Another useful way to assess the relative strength of the demographic variables is via multivariate regression trees. For example, we can identify rule sets that show different combinations of abundance, mortality, and regeneration together. Using chestnut oak as an example (Figure 5A and B), we can identify branches with high combinations of IV and PM. For example, we can see that IV and PM together are relatively higher when elvmax is greater than 774 m in the warm zone (Figure 5B). A similar combination for the cold zone (Figure 5A) involves more rules (sieve10 < 63.79 and tmaysep > 17.01 and elvmax > 489.5 and sieve200 < 42.82 and ph < 5.39 and gsai < 1.2). The multivariate decision trees for the other species are in the Supporting information. It should be borne in mind that multivariate regression trees are inherently more complicated than univariate trees and prone to higher errors. Nevertheless, they point to macropopulations responding to multiple processes and can be an important first step toward further analysis.

## Discussion

It has long been recognized that tree species are genetically diverse and distributed widely over the landscape and hence are differentially adapted to different clines. However, most of the tree species models treat the species as homogenous across geographic and environmental gradients, discounting the numerous heterogeneous adaptive factors that operate. This shortcoming is mainly owing to the fact that it is not easy to delineate these groups using existing methodology.

This study is a step toward overcoming this limitation using available FIA and PHZ data and relatively straightforward statistical approaches to unravel ecological patterns in regional species groups. This type of analysis is especially useful when dealing with uncertainty due to climate change, when meaningful management action is necessary to maintain productivity, diversity, and integrity of forested ecosystems (Hof et al. 2011; Hamann and Aitken 2013). Because tree species can be maladapted due to environmental heterogeneity and evolutionary lag, this variation needs to be taken into account while prescribing actions like managed relocation (McLachlan 2007; McLane and Aitken 2012).

The use of demographic data as a proxy for growth and survival, and PHZ for delineating individual species‐based climatic zones, allowed (1) modeling of patterns of adaptation along clines, (2) assessment of the relative importance of climatic, edaphic, and topographic variables, and (3) derivation of ecologically meaningful rules to extract regional species groups of interest. Because the regional species groups can be identified using environmental rules, they can also be treated as macroscale unit of populations with adaptive potential and subject to further fine‐scale genetic analysis.

It is well recognized that cold tolerance and adaptation of plants are some of the major contributing factors to the differentiation of species along ecoclines (Howe and Aitken 2003; Hawkins et al. 2014). The cold tolerance also broadly defines the range limits of many plant species (Mckenney et al. 2007). PHZ therefore is a convenient and fairly accurate proxy for delineating zones based on average annual extreme minimum temperatures that roughly correspond to the physiological cold tolerance of many species. Also, PHZ can be combined with ecoregions to delineate plant adaptation regions (Vogel et al. 2005; Potter and Hargrove 2012; Bower et al. 2014).

The set of three climatic variables chosen to delineate species groups among and within zones – growing season temperature (*tmaysep*), growing season aridity index (*gsai*), and precipitation of the growing season (*pmaysep*) – capture crucial aspects of growing season temperature and moisture stress as revealed by the variability explained in the analysis (Figure 2). These three variables can be considered important to monitor under future climates as they are tied closely to climatic adaptation in tree species. The environmental heterogeneity responsible for the differences in mean abundances as depicted by the terminal nodes of the decision trees is a rough measure of the fitness landscape (Nagaraju et al. 2013). Even though this measure is somewhat confounded in reality by the “realized niche” of FIA data as opposed to the “fundamental niche,” it is a reasonable assumption because (1) on the whole as the current abundance patterns are correlated with the fundamental niche; (2) the IV is a measure of relativized abundance taking into account the biotic interaction of other tree species in the plot.

Analyzing the species responses between the CLR and WTR highlights the differences in eco‐evolutionary forces between them. The CLR and WTR populations can be treated as statistically independent because they are not likely to have gene flow between them (Stone et al. 2011). All the four species show differentiation with respect to the importance of variables between the CLR and the WTR, with the climate component dominating, especially in the warm zone. Within the climate component, we find that the growing season moisture becomes more important in the WTR (Figure 2). These dynamic patterns revealed by the results show the increasing importance of climate, especially moisture stress in the warm zone under current conditions. This is likely to exacerbate under future climatic conditions where increased temperature and anomalous precipitation changes can easily lead to moisture stress. There is a general pattern that where PM tends to increase in the WTR (Figure 3), there is a corresponding decrease in regeneration. These patterns would have been harder to decipher if the four species were treated as homogeneous across the entire range of each.

Recursive partitioning techniques revealed patterns of univariate and multivariate response/s that can be deciphered from decision tree rules. These were used to extract rules and species groups of interest: in the univariate case, IV, and, in the multivariate case IV, PM, and SC in tandem. Also, rules from macroscale models at 10 km were used to capture populations at finer scale 4‐km data. This feature is useful when lower branches of the tree depict finer scale processes in macroscale models (e.g., climate variables can split the decision tree higher up, but lower down the tree, finer scale soil/topographic features define the splits). We can extend this idea further to hierarchically extract populations. We can, for example, model the geographic space depicted by the node of interest using finer resolution data to unravel processes not evident in the macroscale model.

As abundance, mortality, and regeneration responses will all change under future climatic and disturbance regimes, analyzing them together reveals patterns that are obscured in only univariate analyses, although it should be borne in mind that these can vary due to factors not modeled, such as insect outbreak and ice damage.

### Caveats and features

The insights and patterns gleaned from this study of intraspecific variation should be interpreted with caution, within the context and the purposes of this study, and compared to other studies via corroboration and refutation. First of all, the set of nine predictor variables chosen is deliberately parsimonious for better interpretability and may exclude other variables that could be important in delineating environmental heterogeneity. Also, observed trends can be confounded by other local, modifying factors (ecological interactions) that cannot be modeled adequately (Matthews et al. 2011) and also by limitations due to the spatial and temporal constraints imposed by the data. For example, if moisture is not limiting in the southern warm zone, abundance could increase for some species with future increasing temperatures and higher CO_2_ concentrations (Boisvenue and Running 2006). The patterns of changes in mortality, SC, and abundance are geographic snapshots in time and are the consequence of eco‐evolutionary histories. For example, eastern hemlock currently undergoing decline due to HWA. But these patterns can be misleading if interpreted as changes over time (Lines et al. 2010; Clark et al. 2011; Dietze and Moorcroft 2011). Also, the outputs of the multivariate analysis via regression trees should be treated as an exploratory analysis to assess useful patterns of demography that need to be probed further, not as definitive results.

Another feature of the study is the fairly subjective nature of climatic zone delineations in tune with the macroscale nature of the study. This is inevitable given that there are no clear definitions of how the trees transition geographically. Zone delineations depend on individual researcher's need and the problem at hand and there are likely to be more than three adaptive zones for most species. Because there are no definitive rules for delineating zones, heuristics can be employed to obtain desired results. Fine‐tuning zone delineation with better ecological and genetic information would likely improve results (Wang et al. 2010; Potter and Hargrove 2012; Bower et al. 2014). A further consideration with respect to climatic zones is that the boundaries are not rigid, especially with anticipated rapid climate change. The PHZs are likely to change in the future, as they have in the recent past, based on the global circulation model climate scenarios. This fact needs to be considered when delineating zones under climate change.

Although there are some parallels with this approach and common garden experiments, I do not imply equivalence – because common garden experiments evaluate number of genotypes to quantify the genetic component of phenotypic variation and has far greater applicability in seed zone selection. However, common garden experiments are expensive and not available for many species of interest – therefore, even though relevant, intraspecific variation is not explored in many studies. This approach hopefully opens an avenue to explore intraspecific variation based on widely available demographic and environmental data and provides a method to screen species that have the potential to be explored further via common garden experiments.

I refrained from building predictive models because my main objective was to lay out a methodology for exploring and modeling intraspecific variation in abundance, mortality, and regeneration from easily available data and comparing the differences among and within zones. Therefore, a fairly straightforward nonparametric decision tree ensemble approach that takes into account nonlinearity and interactions was used to derive predictor importance and assess model fit. The presence of hierarchical, nested relationships can be modeled using more sophisticated models to better decipher patterns and processes within and among populations. These models do, however, make large parametric assumptions imposed by the joint distribution of parameters, predictors, and responses, and has its own strengths and limitations that are beyond the scope of the present study (Clark et al. 2014).

In summary, this study highlights the importance of considering intraspecific variation for tree species that span multiple environmental gradients. It provides a methodology for delineating species‐specific climatic zones based on easily available PHZ data and assessing species demography based on FIA's abundance, mortality, and regeneration data as a proxy for overall growth and fitness. Meaningful ecological interpretation using both univariate and multivariate approaches between cold and warm zones is possible using decision tree ensembles and ordination. Further, based on rules derived from these analyses, ecologically meaningful species groups can be identified and extracted for further analysis. The information gained can be used for improving forest management, especially to guide better relocation of vulnerable tree species in this era of rapid climate change and other anthropogenic disturbances.

## Conflict of Interest

None declared.

## Supporting information


**Appendix S1.** Supplementary Material.
