# Supplementary material for: Macroscale intraspecific variation and environmental heterogeneity: analysis of cold and warm zone abundance, mortality, and regeneration distributions of four eastern US tree species
Source: Ecol Evol. 2015 Oct 16;5(21):5033–48. doi: 10.1002/ece3.1752 (PMC4662312; doi:10.1002/ece3.1752)

**Macro-scale intraspecific variation and environmental heterogeneity: analysis of cold and warm zone abundance, mortality and regeneration distributions of four eastern US tree species**

**Anantha M. Prasad**

**Online Supplement**

Table 2. The predictors used in the decision tree based ensemble models. The cell resolution is 10 km for the models and 4 km for mapping decision tree rules.

| **Predictor abbreviation** | **Description** |
| --- | --- |
| *gsai* | Growing season aridity index: Ratio of total May to September precipitation to mean May to September potential evapotranspiration |
| *tmaysep* | Growing season average temperature: May to September average temperature, ^o^ Celsius |
| *pmaysep* | Growing season average precipitation: May to September average precipitation, mm |
| *ph* | Soil pH |
| *sieve10* | Percentage coarse-texture, ( > 2 mm) |
| *sieve200* | Percent fine-texture, (< 0.074 mm) |
| *clay* | Percent clay, (< 0.002 mm) |
| *sprod* | Soil productivity: ordinal measure derived from family-level soil taxonomy information. Soils are ranked from 0 (least productive) to 19 (most productive) based on organic matter content, cation exchange capacity, and percent clay |
| *elvmax* | Maximum elevation in meters, US Geological Survey, 30 m SRTM data |

Figure 4S1. The map corresponding to the rule-sets for cold zone (a) and warm zone (b) for eastern hemlock depicted by the eight-node decision tree. The colors at the terminal nodes are the legend for the map and can be traced by traversing the tree from the top node. The numbers at the terminal node are the mean IV (top) and number of pixels (bottom) for that node. The numbers below the rules in the non-terminal nodes are: mean IV; number of pixels; % deviance explained. The total deviance explained by the pruned decision tree is also shown. See Table 2 for description of the predictors.

a)


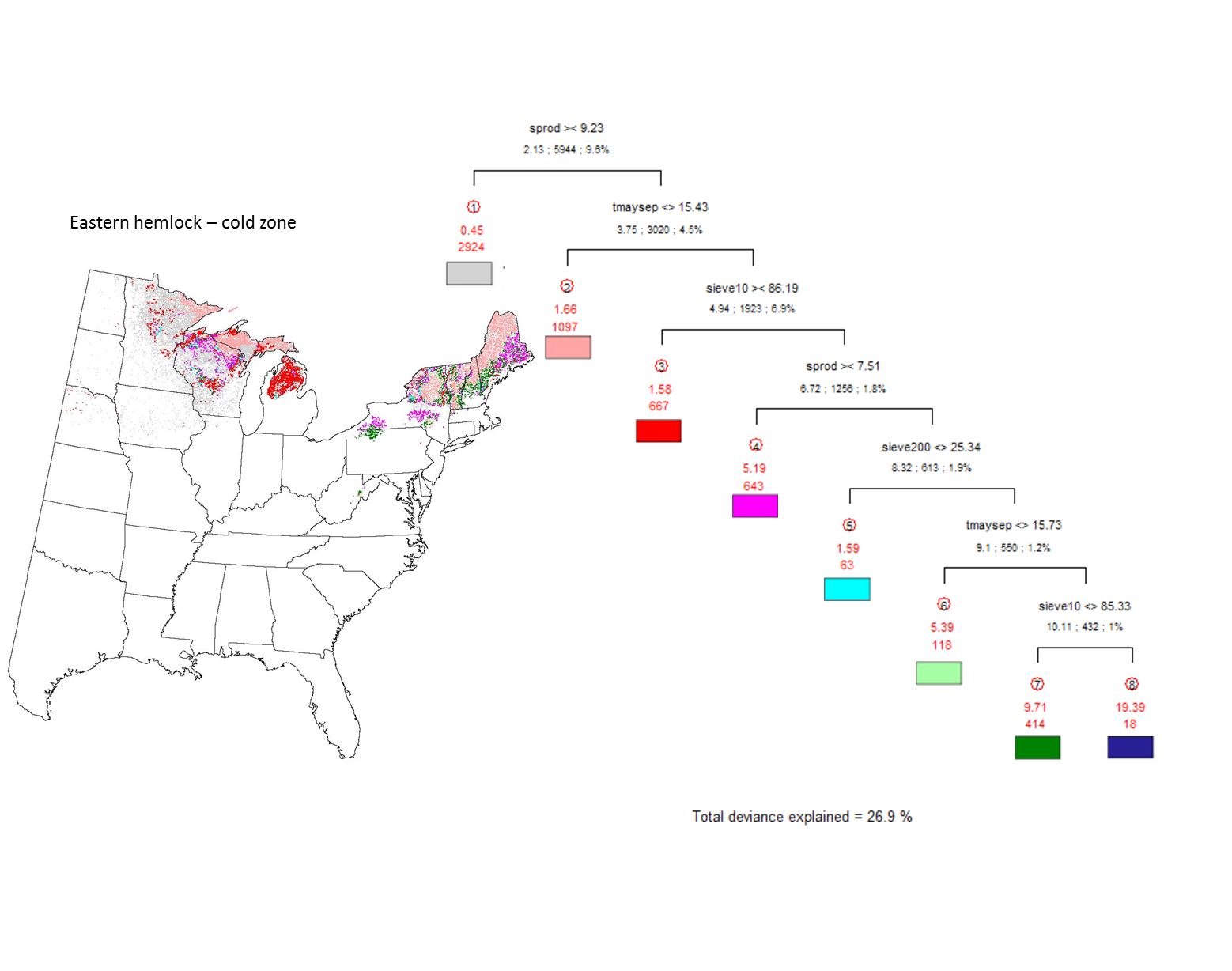


b)
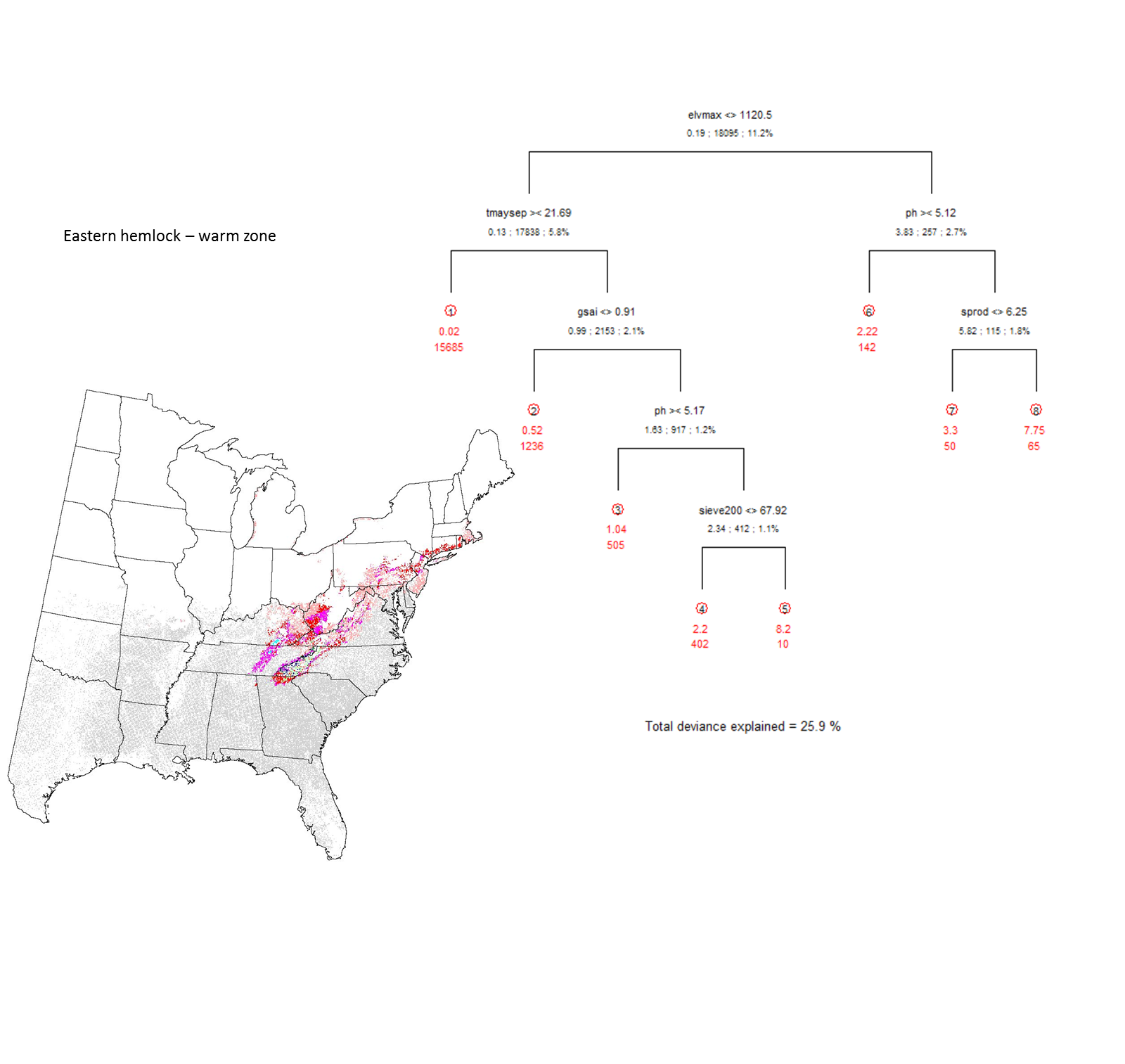


Figure 4S2. The map corresponding to the rule-sets for cold zone (a) and warm zone (b) for tulip poplar depicted by the eight-node decision tree. The colors at the terminal nodes are the legend for the map and can be traced by traversing the tree from the top node. The numbers at the terminal node are the mean IV (top) and number of pixels (bottom) for that node. The numbers below the rules in the non-terminal nodes are: mean IV; number of pixels; % deviance explained. The total deviance explained by the pruned decision tree is also shown. See Table 2 for description of the predictors.

a)


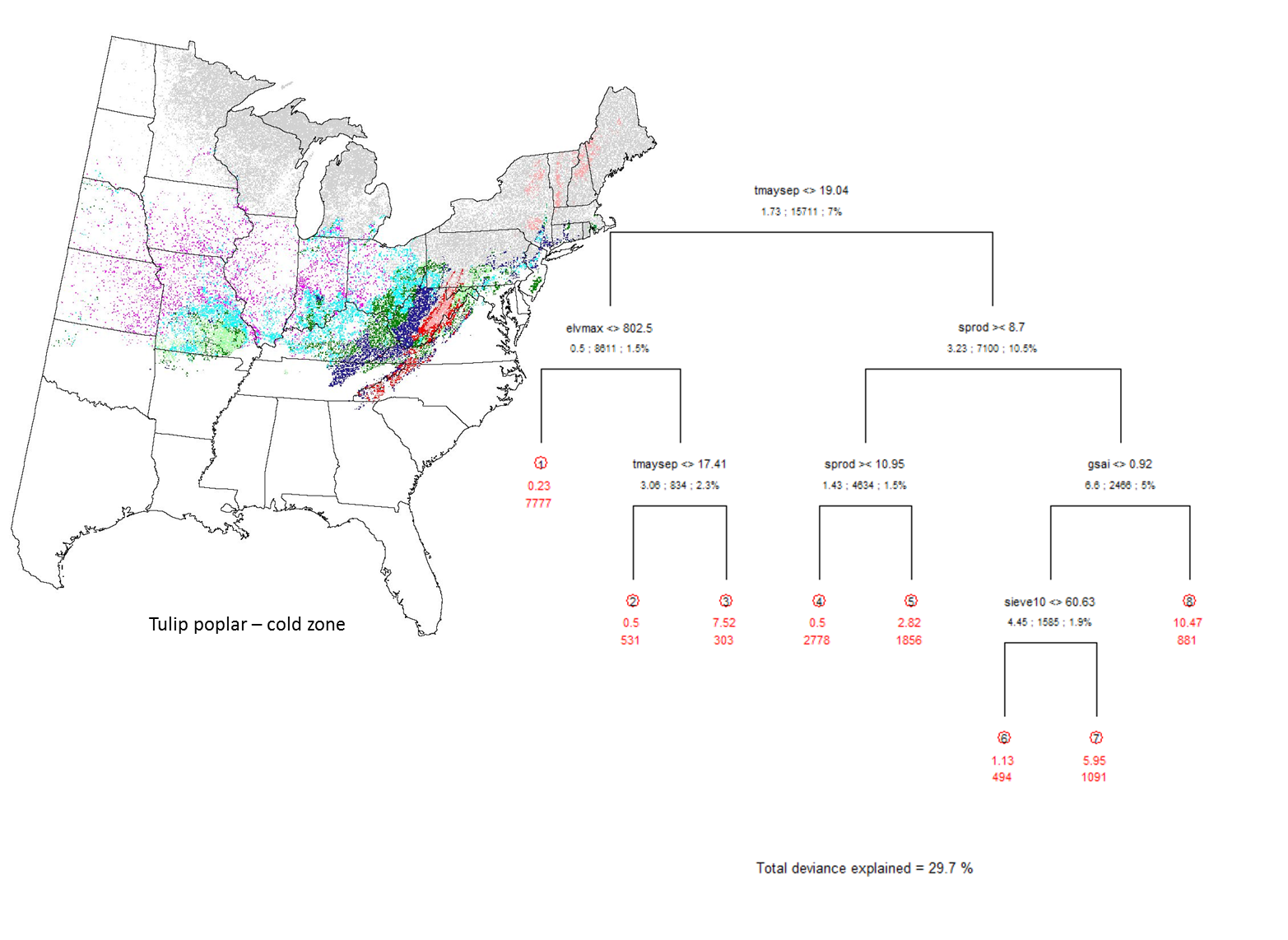


b)


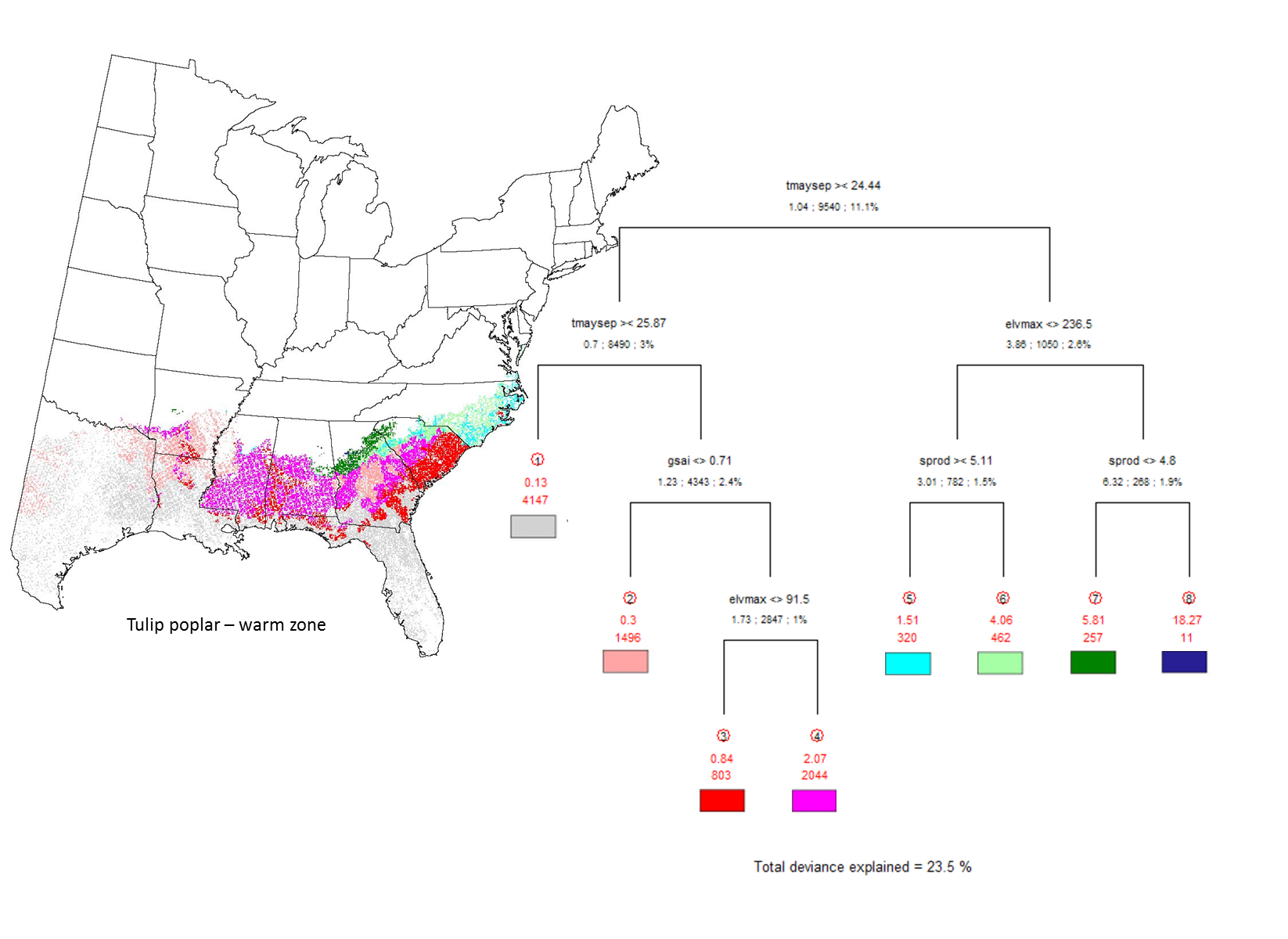


Figure 4S3. The map corresponding to the rule-sets for cold zone (a) and warm zone (b) for chestnut oak depicted by the eight-node decision tree. The colors at the terminal nodes are the legend for the map and can be traced by traversing the tree from the top node. The numbers at the terminal node are the mean IV (top) and number of pixels (bottom) for that node. The numbers below the rules in the non-terminal nodes are: mean IV; number of pixels; % deviance explained. The total deviance explained by the pruned decision tree is also shown. See Table 2 for description of the predictors.

a)


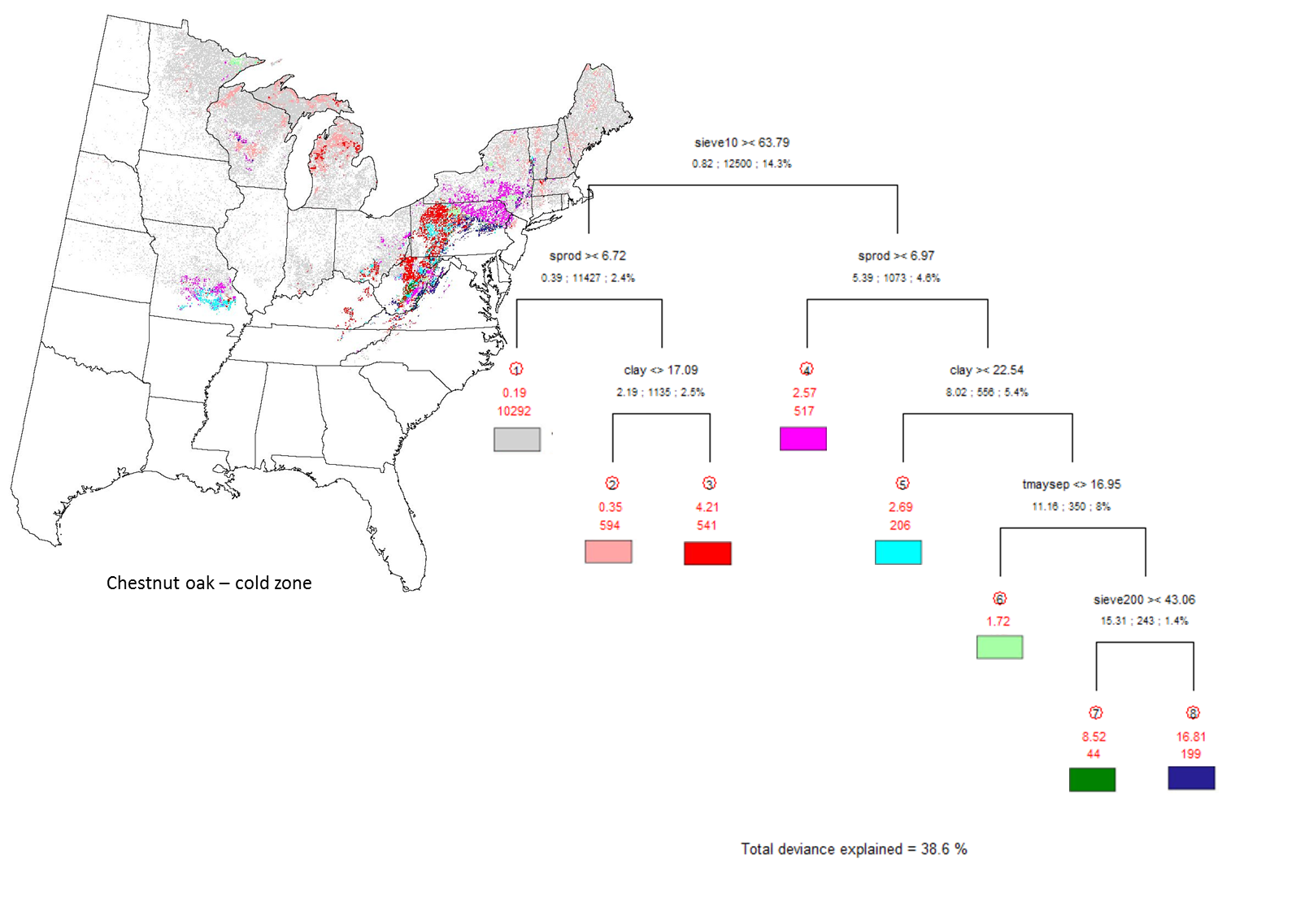


b)


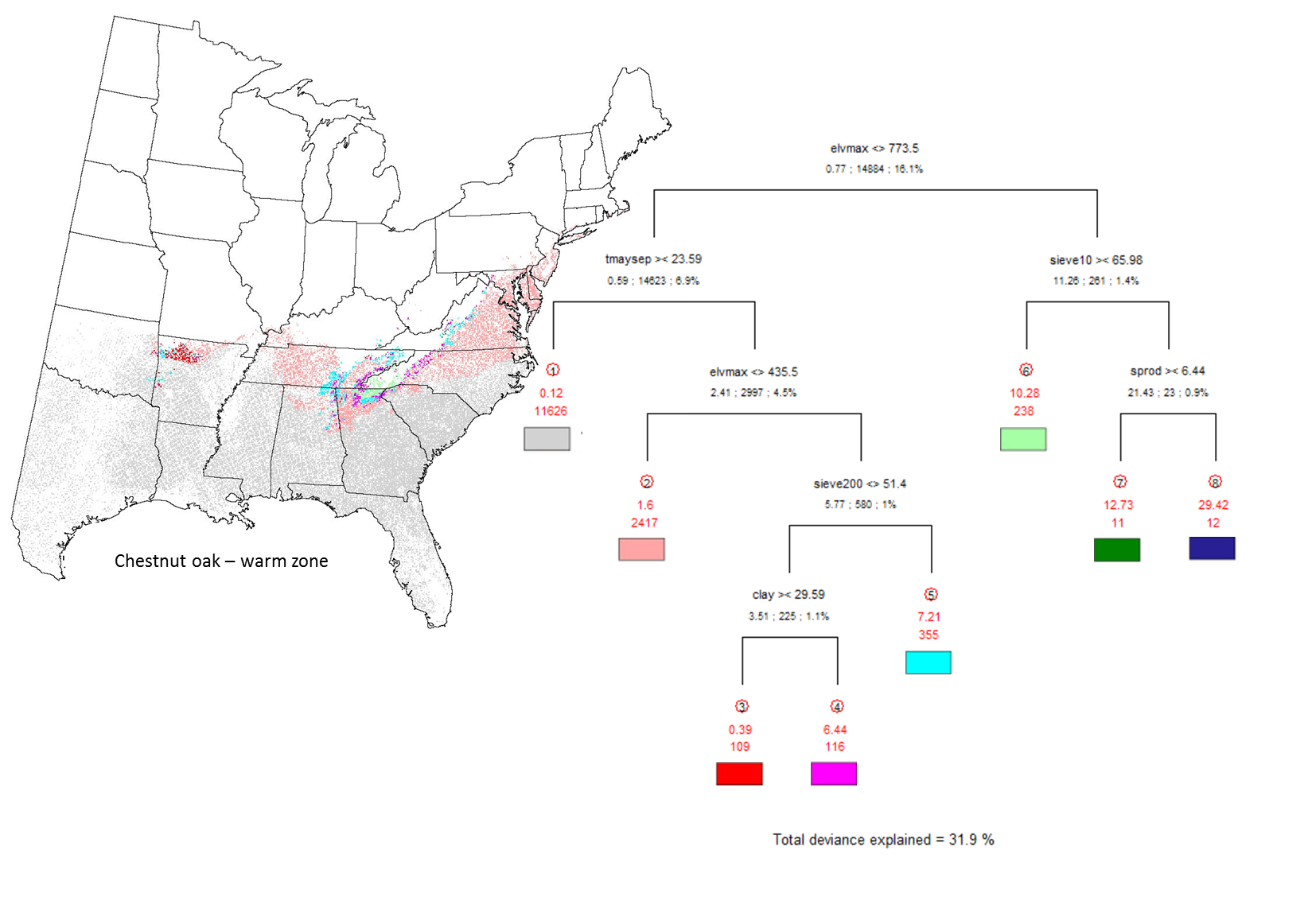


Figure 5S1 The multivariate decision tree for the cold zone (a), and warm zone (b) of eastern hemlock. One can trace the rules that predict different combinations of importance value (IV), percent mortality (PM) and seedling count (SC) together. Under each histogram depicting the multivariate response, n is the number of pixels in the node; the value before n is the sum of squared errors for the node. The length of the lines corresponds to the importance of the branch in explaining the deviation. See Table 2 for description of the predictors.

a)


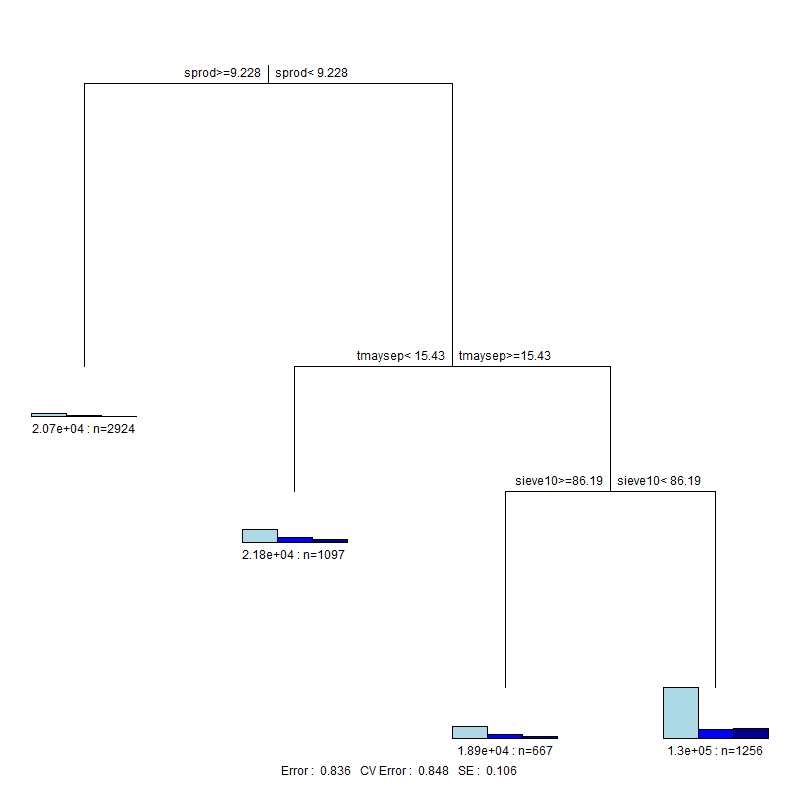


b)


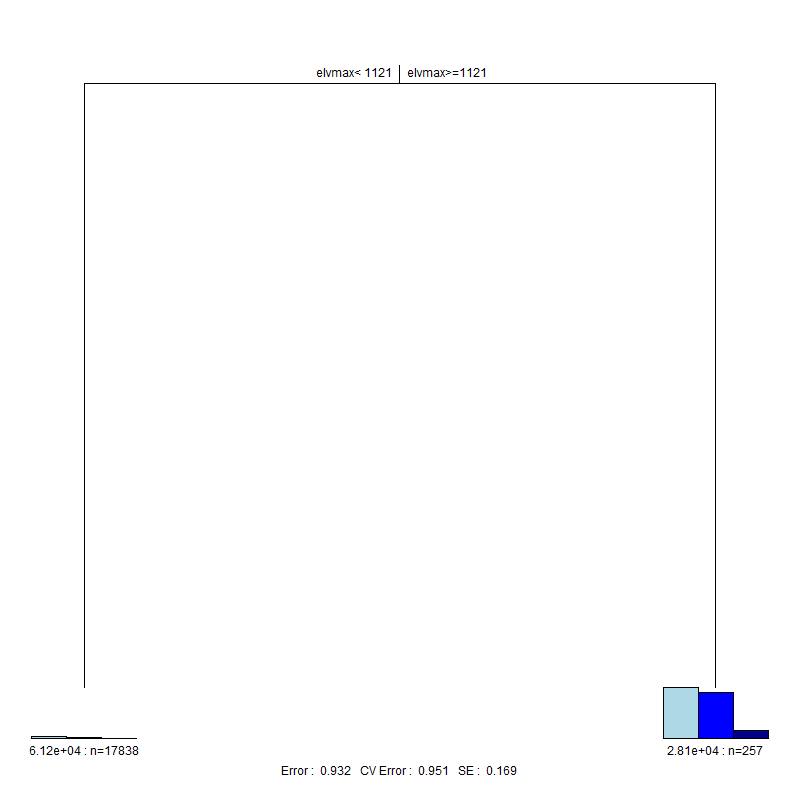


Figure 5S2 The multivariate decision tree for the cold zone (a), and warm zone (b) of sweet birch. One can trace the rules that predict different combinations of importance value (IV), percent mortality (PM) and seedling count (SC) together. Under each histogram depicting the multivariate response, n is the number of pixels in the node; the value before n is the sum of squared errors for the node. The length of the lines corresponds to the importance of the branch in explaining the deviation. See Table 2 for description of the predictors.

a)


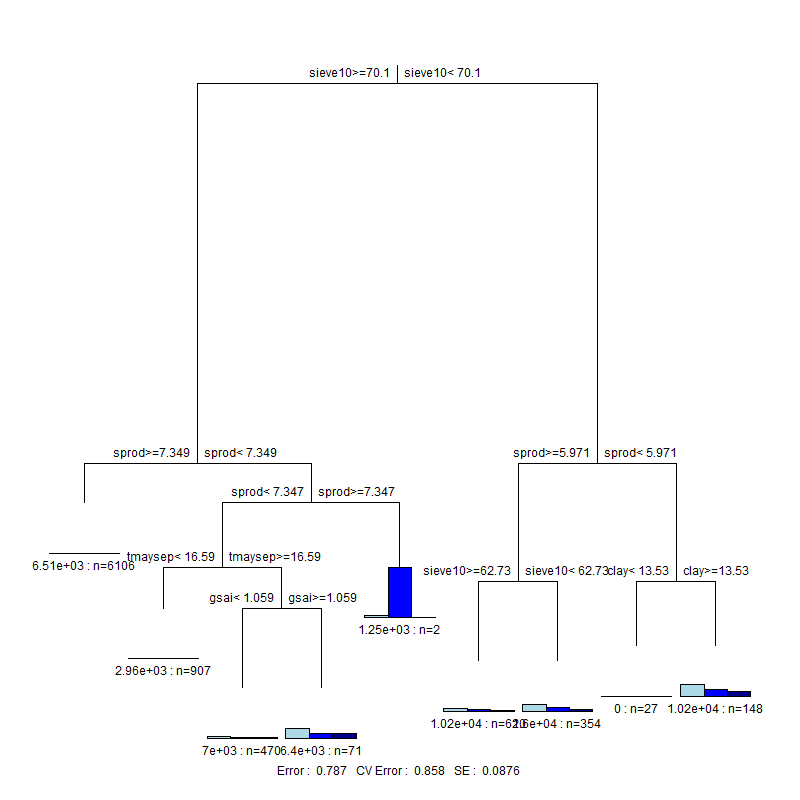


b)


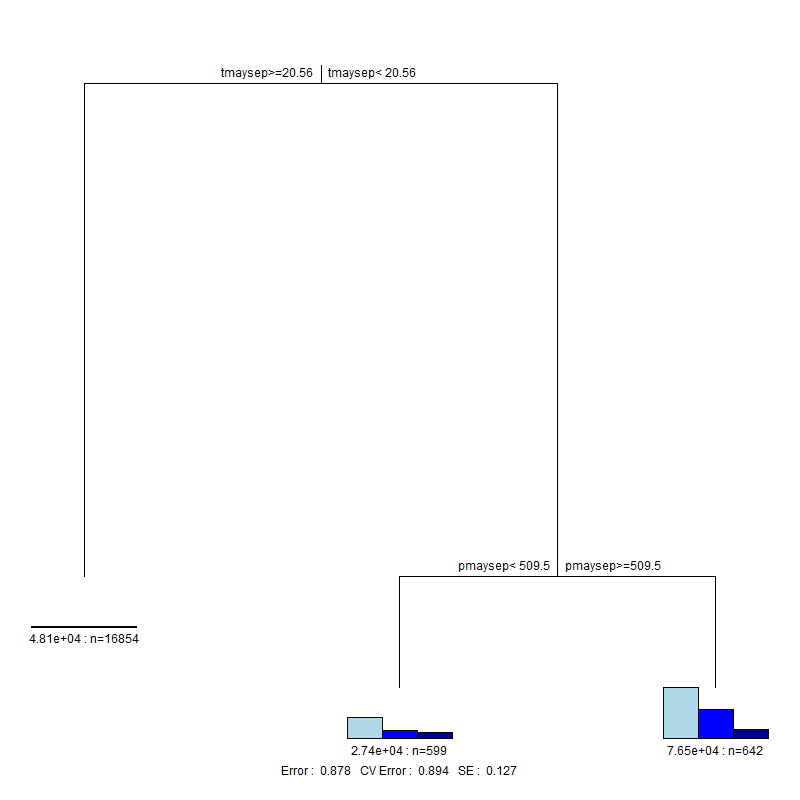


Figure 5S3 The multivariate decision tree for the cold zone (a), and warm zone (b) of tulip poplar. One can trace the rules that predict different combinations of importance value (IV), percent mortality (PM) and seedling count (SC) together. Under each histogram depicting the multivariate response, n is the number of pixels in the node; the value before n is the sum of squared errors for the node. The length of the lines corresponds to the importance of the branch in explaining the deviation. See Table 2 for description of the predictors.

a)


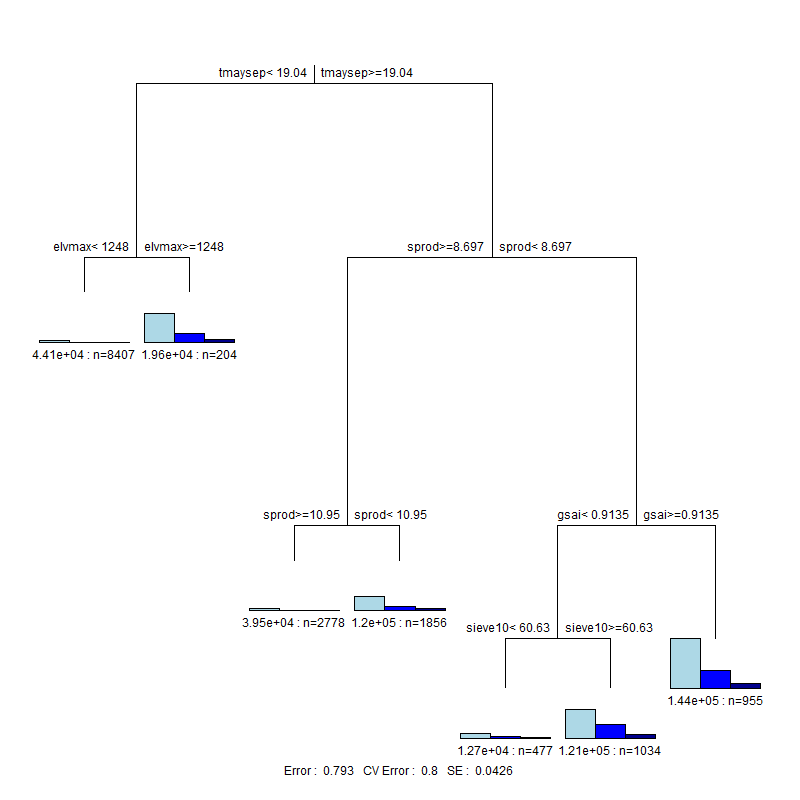


b)


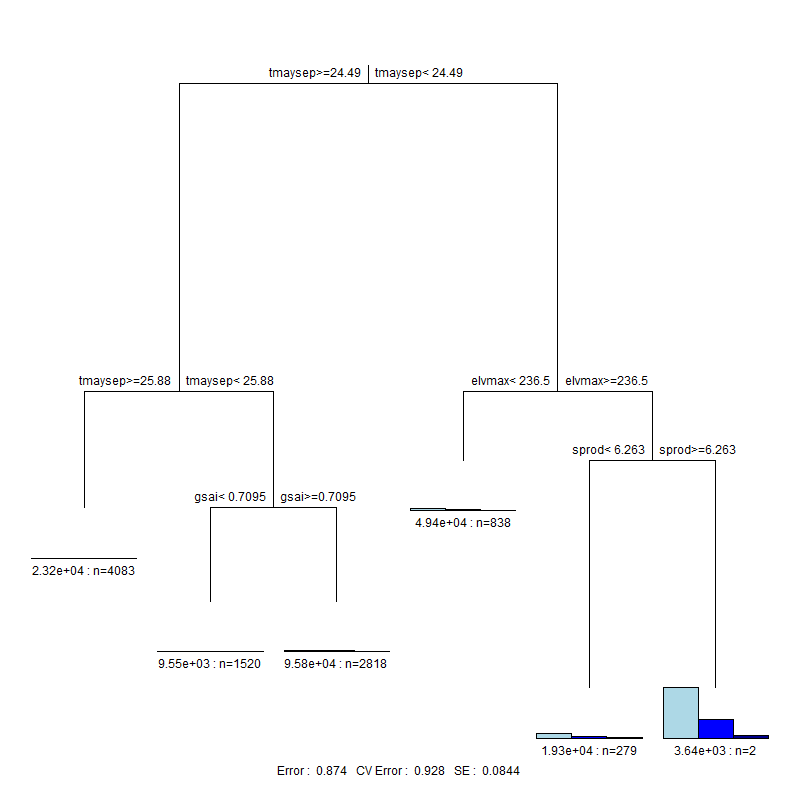

Supplement: Supplementary file 1 — Appendix S1. Supplementary Material. [file ECE3-5-5033-s001.docx]
